# Fluorescence correlation spectroscopy, combined with bimolecular fluorescence complementation, reveals the effects of β-arrestin complexes and endocytic targeting on the membrane mobility of neuropeptide Y receptors

**DOI:** 10.1016/j.bbamcr.2012.03.002

**Published:** 2012-06

**Authors:** Laura E. Kilpatrick, Stephen J. Briddon, Nicholas D. Holliday

**Affiliations:** Cell Signalling Research Group, School of Biomedical Sciences, University of Nottingham, The Medical School, Queen's Medical Centre, Nottingham, NG7 2UH, UK

**Keywords:** BiFC, bimolecular fluorescence complementation, BIBO3304, (*R*)-*N*_2_-(diphenylacetyl)-*N*-[(4-(aminocarbonylaminomethyl-)phenyl)methyl]-argininamide, BSA, bovine serum albumin, DMEM, Dulbecco's modified Eagle's medium, FCS, fluorescence correlation spectroscopy, FRAP, fluorescence recovery after photobleaching, Gc, sfGFP fragment 155–238, Gn, sfGFP fragment 2–173, GPCR, G protein coupled receptor, HBSS, HEPES buffered saline solution, NPY, neuropeptide Y, PCH, photon counting histogram, (sf)GFP, (superfolder) green fluorescent protein, YFP, yellow fluorescent protein, G protein coupled receptor, Neuropeptide Y, Arrestin, Fluorescence correlation spectroscopy, Bimolecular fluorescence complementation, Endocytosis

## Abstract

Fluorescence correlation spectroscopy (FCS) and photon counting histogram (PCH) analysis are powerful ways to study mobility and stoichiometry of G protein coupled receptor complexes, within microdomains of single living cells. However, relating these properties to molecular mechanisms can be challenging. We investigated the influence of β-arrestin adaptors and endocytosis mechanisms on plasma membrane diffusion and particle brightness of GFP-tagged neuropeptide Y (NPY) receptors. A novel GFP-based bimolecular fluorescence complementation (BiFC) system also identified Y1 receptor-β-arrestin complexes. Diffusion co-efficients (*D*) for Y1 and Y2-GFP receptors in HEK293 cell plasma membranes were 2.22 and 2.15 × 10^− 9^ cm^2^ s^− 1^ respectively. At a concentration which promoted only Y1 receptor endocytosis, NPY treatment reduced Y1-GFP motility (*D* 1.48 × 10^− 9^ cm^2^ s^− 1^), but did not alter diffusion characteristics of the Y2-GFP receptor. Agonist induced changes in Y1 receptor motility were inhibited by mutations (6A) which prevented β-arrestin recruitment and internalisation; conversely they became apparent in a Y2 receptor mutant with increased β-arrestin affinity. NPY treatment also increased Y1 receptor-GFP particle brightness, changes which indicated receptor clustering, and which were abolished by the 6A mutation. The importance of β-arrestin recruitment for these effects was illustrated by reduced lateral mobility (*D* 1.20–1.33 × 10^− 9^ cm^2^ s^− 1^) of Y1 receptor-β-arrestin BiFC complexes. Thus NPY-induced changes in Y receptor motility and brightness reflect early events surrounding arrestin dependent endocytosis at the plasma membrane, results supported by a novel combined BiFC/FCS approach to detect the underlying receptor-β-arrestin signalling complex.

## Graphical abstract

**Figure:**
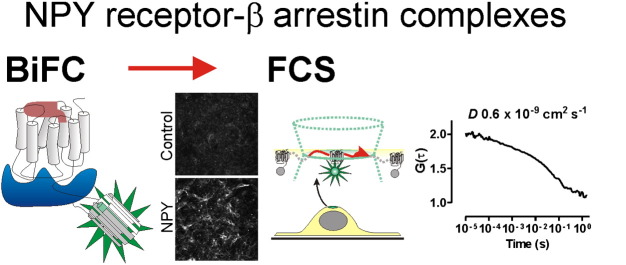

**Highlights:**

► First FCS / PCH analysis of the plasma membrane diffusion characteristics of NPY receptors. ► Demonstration that NPY changes in receptor diffusion and oligomeric state depend on early events in endocytosis. ► Design of a new GFP BiFC system for use in combination with FCS techniques. ► Isolation of the mobility and molecular brightness of defined receptor-arrestin complexes undergoing endocytosis.

## 1 Introduction

G protein coupled receptors (GPCRs) constitute a large and diverse array of cell surface receptors, which respond to signalling molecules ranging from metal ions to large polypeptide hormones. They are now known to activate a wide variety of signalling and regulatory pathways, beyond those mediated by classical heterotrimeric G proteins. Other effector proteins, most prominently the two non-visual isoforms of the β-arrestin family, can interact with the agonist bound GPCR to form alternative complexes [1,2]. β-Arrestins were originally described as simple terminators of G protein signalling, but their roles have rapidly expanded to those of multifunctional adaptors. Their association with clathrin, AP-2 and phosphotidylinositides drives internalisation of many GPCRs, and the stability of the internalised GPCR-β-arrestin complexes can dictate subsequent receptor trafficking to recycling or degradative pathways [2–8]. Moreover they recruit a range of other enzymes to the GPCR which can both regulate G protein dependent events [9,10] and initiate G protein independent signalling, for example through scaffolding of mitogen activated protein kinase cascades [1,2,11]. Structurally, several adaptor binding domains overlap on the β-arrestin surface, suggesting that some β-arrestin-based signalling complexes must form to the exclusion of others [2]. Understanding the spatiotemporal formation of different GPCR-β-arrestin scaffolds is thus an important element in defining how subsequent signalling is orchestrated. Potentially, this also influences the ability of some GPCR ligands to direct these events in a pathway specific manner [1]. Studying this organisation in part requires techniques to investigate such complexes within microdomains of single cells, rather than the overall population response.

Fluorescence correlation spectroscopy (FCS) is an imaging technique that is in theory capable of this resolution in living cells [12–14]. As fluorescent molecules pass in and out of a confocal detection volume fixed in position, they generate time-dependent fluctuations in intensity. Analysis of the fluctuations then provides information about the mobility and concentration of the fluorescent particles. Since the size of the confocal volume is small (~ 0.25 μm^3^), FCS can investigate the properties of fluorescent species within a precise cellular region, for example containing ~ 0.1 μm^2^ plasma membrane. Whilst not a true “single molecule” technique, FCS is highly sensitive and typically reflects the behaviour of 1–100 particles within the detection volume. The same fluctuation records can also undergo a separate statistical analysis. In this case the amplitude variation about the mean intensity is considered, using photon counting histogram (PCH) or fluorescence intensity distribution analysis [15,16]. PCH analysis provides complementary information to FCS, in particular by also estimating the particle brightness of individual fluorescent species. These techniques have been successfully applied in cells to study the plasma membrane diffusion and oligomeric state of GPCRs tagged with variants of green fluorescent protein (GFP) [17–21], and to monitor the binding of fluorescent GPCR ligands [17,22,23].

One of the inherent challenges for FCS and PCH analysis is to correlate changes in observed parameters, such as particle diffusion co-efficients or brightness, with clearly defined signalling events and known molecular GPCR complexes. We and others have recently described one approach to this problem, in which bimolecular fluorescence complementation (BiFC) identifies the association between GPCRs and their partners [18,24–26]. BiFC involves the use of complementary fluorescent protein fragments as fusion tags for the interacting proteins of interest [25–27]. On protein–protein association these fragments (typically of yellow fluorescent protein, YFP) are brought together to allow refolding, chromophore maturation, and thus an indicative fluorescent signal. BiFC is irreversible, for example on association between GPCRs and β-arrestins [24], and thus unambiguously defines the associated complex for investigation. Moreover, the same range of fluorescence methods can be applied to wild type and complemented fluorescent proteins, since their underlying photophysical properties remain very similar [27]. For example, we have recently used FCS to study GPCR dimers identified by BiFC [18]. However a significant limitation of the combined technique has been that FCS measurements are more problematic using YFP tagged proteins. Such measurements are limited by the more complex photophysics and increased photobleaching inherent to these GFP variants [28].

Here we use a combination of FCS, PCH analysis, fluorescence recovery after photobleaching (FRAP) and novel BiFC approaches to study the plasma membrane mobilities and clustering of neuropeptide Y (NPY) Y1 and Y2 receptors. These are widely expressed G_i_-coupled receptors with key roles in processes such as central control of food intake, cardiovascular regulation and bone metabolism [29]. Using GFP-tagged receptors and mutants that enhance or eliminate β-arrestin association and internalisation, we show how NPY-induced changes in receptor diffusion are clearly correlated with activated receptor targeting to arrestin dependent endocytosis mechanisms. We develop a new BiFC system based on a version of superfolder (sf)GFP [30], which for the first time allows study of molecularly defined Y1-β-arrestin complexes by FRAP, FCS and PCH analysis, and demonstrates their slow mobility, multimeric clustering and heterogeneity. Moreover brightness analysis supports a symmetric mode of recruitment of β-arrestins to individual Y1 receptor complexes.

## 2 Materials and methods

### 2.1 Molecular biology

Standard molecular biology reagents were purchased from Fermentas (St. Leon-Rot, Germany), Promega (Southampton, U.K.) or Sigma-Aldrich (Poole, U.K.). Sequential site-directed Quikchange mutagenesis (Stratagene, La Jolla, CA, U.S.A.) generated our version of sfGFP, in which folding mutations M153T and V163A (amino acid numbering refers to wild type GFP), and the two most critical “superfolder” mutations S30R and Y39N [30] were introduced into an enhanced GFP template. PCR was then used to construct sfGFP fragments Gn (2–172) and Gc (155–238). SfGFP, Gn and Gc cDNAs (lacking start Met) were each placed between XhoI and XbaI sites in either pcDNA 4 TO or pcDNA3.1zeo + (Invitrogen, Paisley, U.K.). Rat Y1 and human Y2 receptor cDNAs, with an N terminal FLAG epitope (DYKDDDDK), were inserted upstream in these vectors between KpnI and NotI sites. All receptor fusion proteins thus contained a consistent linker (LRPLE) between the C terminus of the receptor and fused GFP fragment. For transfection, Y1-Gc and Y2-Gc receptor cDNAs were transferred to the neomycin resistant pCMV FLAG vector (Stratagene). Full length human β-arrestin1, β-arrestin2, and the deletion mutant β-arrestin2 ΔLIEFD (amino acids 373–377) were all cloned in pcDNA3.1zeo+, removing the stop codons, to generate constructs fused to Gn at the C terminus (linker QRPLE). Construction of the Y1 6A and Y2 H155P mutants, and YFP BiFC cDNAs has been described previously [24]. A membrane tagged version of sfGFP was generated by the addition of the dual palmitoylated N terminal motif of GAP-43 (ML**CC**MRRTKQVEKNDEDQKILE…) [31] using synthetic oligonucleotide linkers (GAPsfGFP). All cDNAs were fully sequenced to confirm their identities, and primer sequences are available on request.

### 2.2 Cell culture and transfection

HEK293T and 293TR cells (Invitrogen) were cultured in Dulbecco's modified Eagle's medium (DMEM, Sigma-Aldrich) supplemented with 10% foetal bovine serum, and passaged when confluent by trypsinisation (0.25% w/v in Versene). All transient and stable transfections were performed using Lipofectamine in Optimem (Invitrogen, standard protocol). Mixed population 293TR Y1sfGFP and Y2sfGFP cell lines were generated by transfection of receptor cDNAs in pcDNA4 TO and dual selection for the tetracycline repressor protein (blasticidin, 5 μg ml^− 1^) and receptor cDNA (zeocin, 200 μg ml^− 1^). Prior to experiments (18–21 h), receptor expression was induced by 1 μg ml^− 1^ tetracycline treatment. For BiFC experiments, stable HEK293T β-arrestin-Gn clonal cell lines were first generated by zeocin selection and subsequent dilution cloning. Y1 receptor-Gc (in pCMV FLAG) stable mixed populations were then established from selected clonal arrestin-Gn lines by dual G418 (0.8 mg ml^− 1^) and zeocin resistance. All stable transfected cell lines were routinely maintained in DMEM containing blasticidin (5 μg ml^− 1^), zeocin (50 μg ml^− 1^) and/or G418 (0.1 mg ml^− 1^) as appropriate.

### 2.3 [^125^I]PYY competition binding studies

Membranes were freshly prepared from 293TR Y receptor-sfGFP cell lines (after tetracycline induction), or HEK293T Y receptor β-arrestin BiFC cells, as described previously [24]. Competition binding assays were performed for 90 min at 21 °C in buffer (25 mM HEPES, 2.5 mM CaCl_2_, 1.0 mM MgCl_2_, 0.1% bovine serum albumin, 0.1 mg ml^− 1^ bacitracin; pH7.4) and increasing concentrations of unlabelled ligands (0.1 pM–1 μM, duplicate). [^125^I]PYY (Perkin Elmer, Seer Green, U.K.) was used as the radioligand, at 16 pM for Y1 receptors, and at 10 pM for the Y2 subtype. GTPγS displacements also included 30 μg ml^− 1^ saponin in the assay buffer. Membrane bound radioligand was separated by filtration through Whatman GF/B filters soaked in 0.3% polyethyleneimine on a Brandel cell harvester, and retained radioactivity was quantified using a gamma-counter (Packard Cobra II, Perkin Elmer, Waltham, MA, U.S.A.).

Non-specific binding in these experiments comprised less than 5% of total counts, and was subtracted from the data. IC_50_ values were calculated from displacement curves fitted using non-linear least squares regression in GraphPad Prism 5.01 (GraphPad software, San Diego CA, U.S.A.). Hill slopes for these curves ranged from − 0.6 to − 1.1. The Cheng–Prusoff equation converted IC_50_ measurements to pKi values, quoted as mean ± s.e.m. throughout. Homologous PYY displacements were also used to obtain approximate B_max_ estimates (in pmol mg^− 1^ membrane protein) according to B_max_ = TSB × IC_50_/[L], where TSB is the total specific binding in the absence of agonist and [L] is the radioligand concentration.

### 2.4 Quantitative automated imaging of Y receptor-GFP internalisation and arrestin BiFC responses

Cells were seeded at 20 000 cells/well (293TR Y receptor-sfGFP, with tetracycline treatment 24 h later) or at 40 000 cells/well (HEK293 Y1/β-arrestin BiFC stable lines) onto poly-L-lysine coated 96 well black clear bottomed plates (655090, Greiner Bio-One, Gloucester, U.K.). Experiments were performed once cells reached confluence at 24 h, directly after seeding (BiFC cells), or after tetracycline induction of Y receptor-sfGFP expression. Medium was replaced with DMEM/0.1% bovine serum albumin (BSA) and NPY (Bachem, St. Helens, U.K.) was then added for the times indicated (0.1 nM–3 μM, triplicate wells). For reversibility experiments, cells were then washed with DMEM/0.1% BSA (2 × rinse, 1 × 60 min at 37 °C) as detailed in the Results and discussion section. Incubations were terminated by fixation with 3% paraformaldehyde in phosphate buffered saline (PBS, 10 min at 21 °C), the cells were washed once with PBS and the cell nuclei were stained for 15 min with the permeable dye H33342 (2 μg ml^− 1^ in PBS, Sigma). H33342 was then removed by a final PBS wash. Images (4 central sites/well) were acquired automatically on an IX Ultra confocal platereader (Molecular Devices, Sunnyvale, CA, U.S.A.), equipped with a Plan Fluor 40 × NA0.6 extra long working distance objective and 405 nm/488 nm laser lines for H33342 and sfGFP excitation respectively.

An automated granularity algorithm (MetaXpress 2.0, Molecular Devices) identified internal fluorescent compartments within these images of at least 3 μm diameter (range set to 3–18 μm). For each experiment, granules were classified on the basis of intensity thresholds which were set manually with reference to the negative (vehicle) or positive (1 μM NPY) plate controls. The quantified granule parameters for each image (count, area, average intensity) were normalised to number of cells on the image by counting H33342 stained nuclei. Each individual data point was obtained from assessment of 12 images (4 sites/well in triplicate), and normalised as a percentage of the basal controls (set to 100%), to allow pooling of individual experiments. NPY concentration response curves, or one phase association timecourses were fitted to the pooled data by non-linear least squares regression (GraphPad Prism).

### 2.5 Confocal microscopy and FRAP analysis

Transfected cells were grown for 24–48 h in 8 well Nunc Labtek chambered coverglasses coated with poly-L-lysine (Fisher, Loughborough, U.K.), including tetracycline pretreatment as appropriate, to approximately 80% confluence. Cells were imaged at 37 °C in HEPES buffered saline solution (HBSS/0.1% BSA) before and after incubation with NPY. Images were acquired on a Zeiss LSM 510 M laser scanning microscope (Zeiss, Jena, Germany) using a 63 × Plan-Apochromat NA1.4 oil objective, and a pin hole diameter of 1 Airy unit. Ar 488 nm (GFP, Gn/Gc BiFC) laser lines were used for excitation, and emitted light collected via a 505/550 nm bandpass filter. Equivalent laser power and gain settings were used for images of control and agonist treated cells within the same experiment. Identical linear adjustments to contrast and brightness were made to representative images in the figures for presentation purposes.

FRAP experiments (using cells prepared as above) were instead performed on a Zeiss LSM 710 M laser scanning microscope using a 63 × Plan-Apochromat NA 1.4 oil objective. Ar 488 nm (GFP, Gn/Gc BiFC) laser lines were used for excitation and emitted light collected between 493 and 558 nm. Vehicle and 100 nM NPY pretreatments were carried out in HBSS/0.1% BSA at 37 °C for the indicated times, after which the slide was transferred to the heated stage and left to equilibrate at 37 °C. Cell images (512 × 512 pixels) were acquired of the lower plasma membrane adjacent to the coverglass. 10 images (at 0.5 s intervals) were acquired before a 1.4 μm^2^ circular region of interest (ROI, radius r = 0.66 μm) was bleached by 50 iterations of 100% laser power. FRAP was subsequently monitored for 90–120 s. In addition to a background ROI, a representative (adjacent cell) ROI monitored the amount of non-bleached fluorescent signal lost during imaging. FRAP recovery curves were fitted to data corrected for background and representative ROIs (Zen 2010 software, Zeiss, Jena, Germany), using a one phase exponential I(t) = I0 − I1.e^− t/T1^, In this equation I0 represents the end value of recovered fluorescence intensity, I1 is the amplitude of the recovered fraction, and T1 is related to the recovery half-time t_1/2_ by t_1/2_ = − T1.ln 0.5. The mobile fraction percentage was estimated by F1 = 100 × I1 / (IB − IA), where IB is the initial fluorescence intensity and IA is the intensity immediately post bleach. Diffusion coefficients (*D*) were also calculated using the equation *D* = r^2^ / 4 t_1/2._ Pooled data are presented as the mean  ± s.e.m. for *n* cells, with the number of experiments also indicated where appropriate.

### 2.6 Fluorescence correlation spectroscopy and PCH analysis

The FCS method has been described in detail previously [14,17]. Briefly, cells were seeded onto poly-L-lysine coated Nunc Labtek 8-well chambered coverglasses as for confocal microscopy experiments, with receptor induction by tetracycline as appropriate. Vehicle, the non-peptide antagonist BIBO 3304 (1 μM, a gift from Boehringer–Ingelheim GmbH (Biberach, Germany), [32]) and 100 nM NPY pretreatments at 37 °C were carried out in HBSS/0.1% BSA for the indicated times, after which the slide was transferred to the microscope stage (Zeiss Confocor 2, equipped with a c-Apochromat 40 × NA1.2 water-immersion objective) and allowed to equilibrate to 22 °C. For each recording, the confocal volume was positioned on the cell nucleus in x–y, selecting fluorescent cells of approximately equivalent brightness by using a constant camera exposure time for all experiments in this study. A subsequent vertical scan in z identified and located the volume on the upper plasma membrane (see Fig. 2 for illustration). Fluctuations were recorded using 488 nm excitation (2 × 15 s reads), following a 15 s pre-bleach, using a constant laser power (0.61 kW/cm^2^) for all experiments.

Autocorrelation analysis was performed and fitted using Zeiss AIM 4.2 software. A minority of autocorrelation curves (at most 35% total records) were excluded from analysis if decay to a clear asymptote (G(τ) = 1.0) was not observed with increasing time offset τ. A 2D diffusion model, with two components and a pre-exponential term to account for fluctuations caused by photophysical events within GFP (1–2 μs) [14,17], was sufficient to fit autocorrelation data, as assessed by the fit residuals (see Fig. 2). The fits provided apparent dwell times τ_D1_ and τ_D2_ for each component, and its percentage contribution to the overall amplitude of the autocorrelation curve, G(0) (%τ_D1_, %τ_D2_). As discussed later (Section 3.2), we interpreted τ_D1_ as a further component generated by GFP blinking states. Thus the particle number *N* was derived from its inverse relationship to the amplitude of the τ_D2_ component. In all experiments calibration was first carried out by calculating the dwell time of 10 nM Rhodamine 6 G (Invitrogen, *D*_*R*6G_ 2.8 × 10^− 6^ cm^2^ s^− 1^), which allowed estimation of the confocal volume and waist radius (ω_0_ = (4*D*_*R6G*_.τ_D_)^1/2^). The diffusion co-efficient for fluorescent receptor species was then estimated from individual τ_D2_ values by *D =* ω_0_^2^/4τ_D2_, and particle concentration in the membrane by *N*/(πω_0_^2^). In a subset of experiments, PCH analysis was also performed on the raw fluctuation data using Zen 2010, fitting to single component or two component PCH models [33] to obtain estimates of particle number (*N*) and brightness (ε). A bin time of 1 ms was chosen throughout to exclude the time-dependent τ_D1_ fluctuation components assigned to fluorescent protein photophysics, whilst retaining slower fluctuations derived from particle diffusion (τ_D2_). A first order correction factor (based on Rhodamine 6G calibration) was also applied in the analysis to account for deviation from a Gaussian observation volume when using single-photon excitation [33].

Pooled data are presented as the mean ± s.e.m. for *n* cells, with the number of experiments also indicated where appropriate. Differences between multiple data groups were assessed for significance by non-parametric Kruskal–Wallis test, followed by Dunn's post test (GraphPad Prism v5.01).

## 3 Results and discussion

### 3.1 Differential Y1 and Y2 receptor internalisation is identified by automated imaging and analysis

NPY Y1 and Y2 receptors both undergo agonist-promoted internalisation after β-arrestin mediated recruitment to clathrin coated pits [5,24,34–37], but the relative extent of Y2 receptor endocytosis has proved controversial [24,34,36,37]. Here we first determined the concentration-dependence of NPY induced internalisation for C terminal superfolder (sf) GFP tagged Y1 and Y2 receptors expressed in 293TR cells, each under the control of a tetracycline inducible promoter. For each construct we verified that receptor affinities for PYY and NPY were unaltered by the C terminal fusion to sfGFP, measured by competition [^125^I]PYY binding studies (Table 1) — as previously reported [24,38,39]. We confirmed that [^125^I]PYY binding was inhibited by GTPγS, which disrupts the formation of the high affinity complex between agonist bound Y receptor and its coupled G protein (Table 1) [24]. Receptor expression levels (B_max_ 1.5–2.2 pmol mg^− 1^ membrane protein) were also broadly equivalent in all Y receptor-sfGFP cell lines investigated (Table 1).

**Fig. 1:**
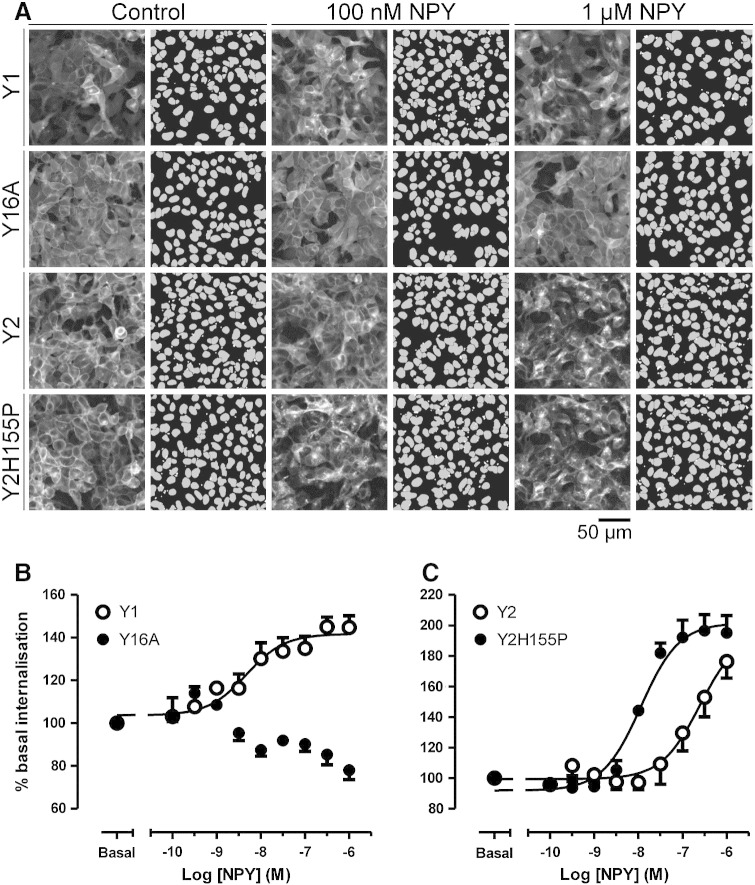
Internalisation of native and mutated Y receptors quantified by granularity analysis. Y1 and Y2 receptors, fused to GFP, were each stably transfected in 293TR cells, with inducible receptor expression initiated by 18 h pretreatment with tetracycline (1 μg ml^− 1^). Representative example experiments (A) show images acquired by the IX Ultra confocal platereader, of cells under control conditions, or treated for 30 min with NPY at the indicated concentration. For each case, the right hand panel indicates the associated granularity analysis, which identified cells from the parallel image of H33342 stained nuclei (grey, original image not shown), together with punctate vesicular structures more than 3 μm in diameter (white dots). Internalisation was quantified as the average granule intensity/cell, normalised as a percentage of the basal measurements, and pooled to provide the concentration − response curves (*n* = 4) shown in (B) and (C). EC_50_ values are quoted in the text.

Y1sfGFP and Y2sfGFP expressing cells were imaged on an IX Ultra confocal platereader (Fig. 1), and their internalisation quantified by automatic identification of receptor-GFP immunofluorescence in intracellular compartments, through granularity analysis. After 15 min treatment, NPY stimulated Y1sfGFP receptor internalisation with a pEC_50_ of 8.32 ± 0.25 (*n* = 4), but was 52 fold less potent in eliciting Y2sfGFP endocytosis (pEC_50_ 6.61 ± 0.22, *n* = 4, *p* < 0.01). Given the increased NPY affinity for Y2 *versus* Y1 receptors in our data (Table 1) and previous studies [5,24,34], our observations are consistent with a requirement for relatively high receptor occupancy by agonists (*e.g*. at μM NPY concentration) to elicit Y2 receptor endocytosis [34,36]. This is also a direct downstream correlate of the reduced agonist potency observed for Y2 receptor recruitment of β-arrestins compared to the Y1 subtype [5,24,39].

The link between β-arrestin recruitment and internalisation was further demonstrated by examining Y1 and Y2 receptor mutants which manipulate β-arrestin interaction specifically. In the Y16A mutant, six serine/threonine residues in the C tail were mutated to alanine (S352A, T353A, T356A, S359A, T361A, S362A) [24]. Removal of these phosphorylation sites was sufficient to eliminate Y1 receptor association with both β-arrestin1 and β-arrestin2 when stimulated with NPY (Supplementary Fig. 1 and Ref. [24]), and also Y16AsfGFP internalisation (Fig. 1). A corresponding difference was observed when following the time course of 100 nM NPY internalisation using live cell confocal microscopy (Supplementary Videos 1 and 2 for Y1sfGFP and Y16AsfGFP receptors respectively). In contrast the H155P mutation enhanced Y2 receptor β-arrestin recruitment (Supplementary Fig. 1 and Ref. [24]), via reconstruction of a second intracellular loop motif important for β-arrestin recognition of activated GPCRs [5,24,40]. The potency of NPY-induced Y2H155P-GFP internalisation was significantly enhanced compared to control Y2 responses (Fig. 1; pEC_50_ 7.94 ± 0.10, *n* = 4; *p* < 0.01), with equivalent cell surface localisation under basal conditions.

### 3.2 FCS and FRAP reveal slowed plasma membrane diffusion of NPY stimulated Y receptors which recruit β-arrestins and internalise

**Fig. 2:**
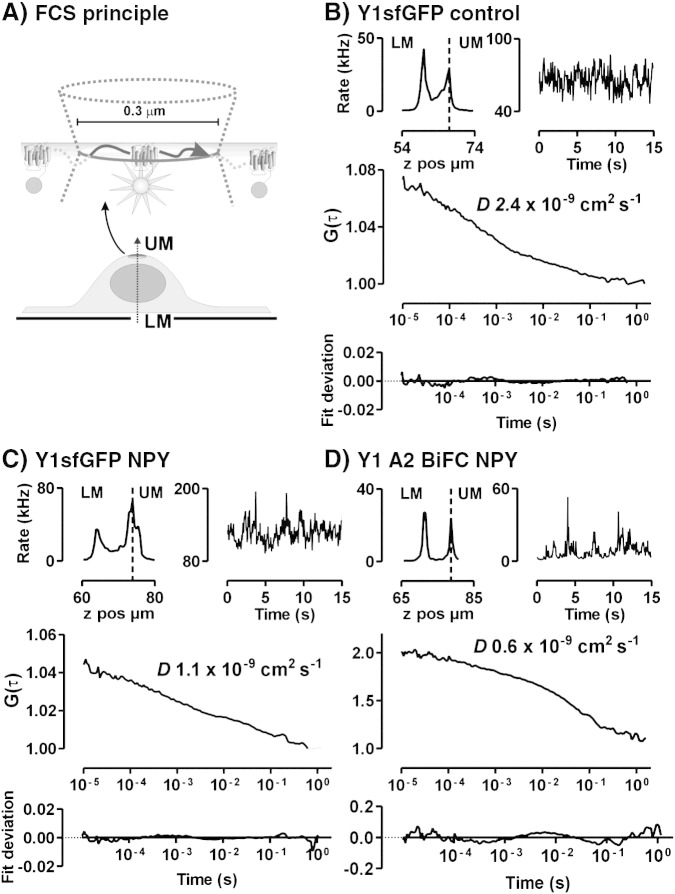
Measuring the lateral mobility of sfGFP-tagged Y receptors and Y receptor-arrestin BiFC complexes by FCS. A schematic diagram of the FCS method is shown in (A), together with three illustrations of FCS recordings and analysis from 293TR Y1sfGFP cells under control conditions (B) or pretreated with 100 nM NPY (C) for 15 min at 37 °C, and from HEK Y1 A2 cells pretreated with 100 nM NPY for 60 min at 37 °C (D). A confocal z-scan, located on the cell nucleus in *x–y*, identified the upper and lower plasma membrane (LM, UM), shown by the peaks in the top left insets for B–D. Following a 15 s prebleach, fluorescence intensity fluctuations were recorded at 22 °C from the confocal volume positioned on the upper membrane (one 15 s read illustrated in top right graphs for B–D), using the same laser power in all cases. The main graphs illustrate the resultant autocorrelation curves, and below, the fit deviation from a two dimensional, 2 component diffusional model (see also Materials and methods). From this model the dwell time for the receptor species was estimated, and thus the indicated value for diffusion co-efficient *D* was derived on the basis of the calibrated confocal volume. Pooled data for these and other parameters are presented in Figs. 3 and 7, and also Tables 2 and 3.

We examined whether the different endocytic behaviours of native and mutant Y1sfGFP and Y2sfGFP receptors were reflected in their lateral mobility on the cell surface, using FCS. Fluorescence fluctuation recordings were generated by diffusion of Y receptor-sfGFP species through the confocal volume positioned on the upper plasma membrane, illuminating an area of ~ 0.1 μm^2^ (Fig. 2A) [13]. Autocorrelation analysis assessed the time-dependence of the fluctuations and provided (i) information about the number of mobile fluorescent particles in the volume (*N*), inversely proportional to the autocorrelation curve amplitude, and (ii) the average particle dwell time (τ_D_), measured at the midpoint of the curve decay and from which the diffusion coefficient *D* was calculated. In practice fitting to an appropriate model isolated individual parameters for multiple additive diffusional components present in the curve [13].

Fluorescence recordings were initially made at 22 °C from the upper plasma membrane of unstimulated 293TR Y1sfGFP cells (Fig. 2). The reduced temperature, rather than a 37 °C environment, is a necessary compromise for obtaining a sufficient number of stable fluctuation measurements, by minimising artefacts from membrane and cell movements. Autocorrelation curves from the intensity fluctuations were adequately fitted to a two dimensional model, with two diffusional components in approximately equal proportion (Table 2). We interpret the first component, with a short apparent dwell time (τ_D1_) of 150–250 μs, as a consequence of the photophysics of the GFP protein [12,17,18,21]. Estimates of the diffusion coefficient for Y1sfGFP receptors were thus derived from the second dwell time τ_D2_ (Table 2). The observed mean value of *D*, 2.22 ± 0.15 × 10^− 9^ cm^2^ s^− 1^ (*n* = 148, from 16 expts), is consistent with other estimates for GPCR diffusion made both by FCS [17–23,28] and fluorescence recovery after photobleaching [41–44] — and indeed, with the membrane mobility of transmembrane proteins in general [43,45]. However it was slower than the diffusion co-efficient of 4.33 ± 0.32 × 10^− 9^ cm^2^ s^− 1^ (*n* = 35, 3 expts) observed for palmitate anchored sfGFP (GAPsfGFP, [31]) in transiently transfected HEK293 cells. It should be noted that individual *D* estimates for Y1sfGFP motility ranged from 0.60 to 15.36 × 10^− 9^ cm^2^ s^− 1^, although 90% values fell within 1.02–3.58 × 10^− 9^ cm^2^ s^− 1^. This heterogeneity might be predicted from sampling small membrane regions, which contain variable proportions of GPCR scaffolding and signalling elements [13,19,21] and which could also be subject to the influence of other factors, such as variations in local membrane topography [46].

**Fig. 3:**
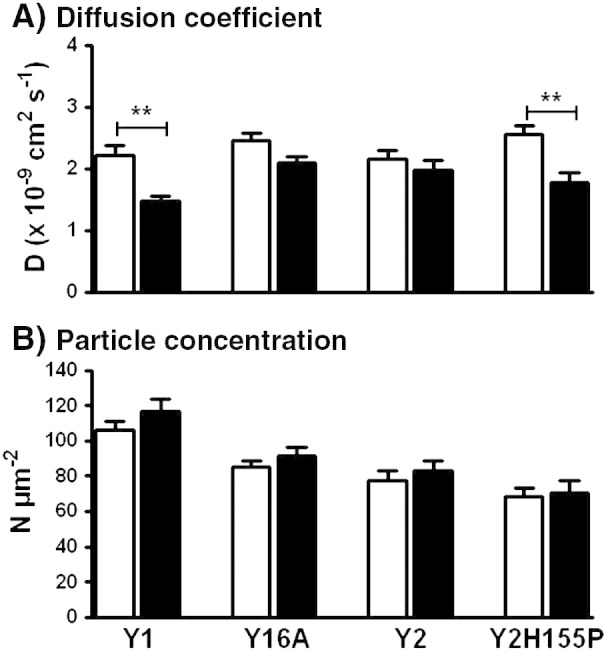
Agonist treatment slows lateral diffusion of Y receptors which undergo rapid endocytosis. Histograms compare pooled data for diffusion co-efficients (A) and particle concentrations (B) from FCS experiments in 293TR cells induced to express Y1sfGFP (*n* = 117–148 cells), Y16AsfGFP (*n* = 87–95), Y2sfGFP (*n* = 47–50) and Y2H155PsfGFP (*n* = 35–51) receptors. Open bars represent vehicle treated cells, whilst solid bars represent measurements made after 15 min NPY (100 nM) pre-treatment at 37 °C. Significant differences between control and NPY treated groups are indicated by ** *p* < 0.01 (Kruskal–Wallis, followed by Dunn's post test).

293TR Y1sfGFP cells were then pretreated with 100 nM NPY for 15 min at 37 °C before FCS observations were made at room temperature (Fig. 2C). For both control and NPY stimulated data sets measurements of *D* and particle concentration did not show time dependence over the room temperature recording period (Supplementary Fig. 2) and were therefore pooled. As before, initial treatment with NPY at 37 °C elicited Y1sfGFP internalisation, with a reduced steady state plasma membrane receptor population then available for FCS measurements. The concentration of Y1sfGFP particles detectable by FCS did not change after agonist exposure, but NPY stimulation significantly slowed Y1sfGFP receptor mobility (Fig. 3, Table 2). To our knowledge these are the first FCS experiments based on an sfGFP fluorescent protein variant, and so we confirmed that similar measurements for τ_D1_, τ_D2_ and *D* were obtained in 293TR cells expressing the Y1eGFP receptor (particle concentrations 63.7 ± 4.2 μm^− 2^ and 62.3 ± 5.0 μm^− 2^ for control and NPY respectively, *n* = 53/46), under identical acquisition conditions (Table 2). This would be expected given that additional superfolder mutations enhance GFP folding kinetics, and do not alter chromophore photophysics [30].

The specificity of the NPY response in 293TR Y1sfGFP cells was investigated using the non-peptide Y1 receptor antagonist BIBO3304 [32], which displaced [^125^I]PYY binding from Y1sfGFP receptors with a pK_i_ of 9.25 ± 0.11 (*n* = 3). BIBO3304 (1 μM) pretreatment (15 min, 37 °C) did not alter the plasma membrane localisation of Y1sfGFP receptors (data not shown), or significantly change their membrane mobility (*D* 2.32 ± 0.22 × 10^− 9^ cm^2^ s^− 1^, *n* = 28 from 4 experiments). However BIBO3304 pretreatment inhibited the effect of 100 nM NPY in decreasing Y1sfGFP receptor diffusion rates (Supplementary Fig. 3).

Our observation that NPY occupancy slowed the diffusion of Y1sfGFP receptors is consistent with FCS studies on the YFP-tagged complement C5a receptor [21], and the identification of both fast and slow diffusing fluorescent agonist species when bound to GPCRs [22,23]. Other investigations, for example on the A_1_ adenosine receptor, have shown no effects of agonist on receptor diffusion [18,20]. The extent to which GPCRs undergo endocytosis provides one predictive element for agonist stimulated reductions in GPCR motility [18,21], but there are notable exceptions — for example the unchanged/increased diffusion rates of the μ-opioid receptor in response to agonists in FCS or FRAP analysis [20,41]. We therefore tested for a link between Y receptor mobility and endocytosis by comparing the diffusion characteristics of the Y1 and Y2 receptors, with the Y16A and Y2H155P mutants. 100 nM NPY pretreatment was used throughout, to maximise the difference in internalisation behaviour between wild type and mutant receptors (Fig. 1). NPY did not alter the diffusion rate of Y2 receptors, but a significant agonist-induced decrease in lateral mobility was restored by introduction of the H155P substitution. Equally the 6A mutation substantially attenuated NPY mediated reductions in Y1sfGFP diffusion (Fig. 3A).

**Fig. 4:**
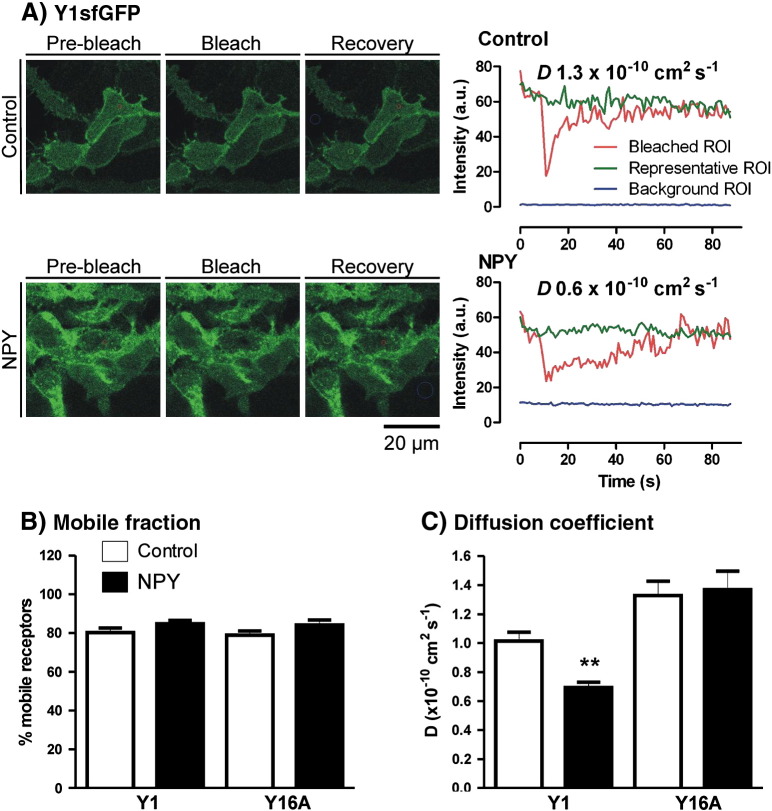
FRAP analysis of Y1sfGFP receptor diffusion. Panel (A) illustrates a representative FRAP experiment performed on the lower plasma membranes of 293TR Y1fGFP cells, under control or NPY (100 nM, 15 min) stimulated conditions. Example cell images show pre-bleach, immediately post bleach and following the 90 s recovery period. To the right fluorescence intensity traces (in arbitrary units, a.u.) are shown over time from the colour coded regions of interest indicated in each recovery image. The data were fitted to a single phase exponential recovery (Zeiss Zen 2010 software, see Materials and methods) to provide estimates of *D* and mobile fraction. Panels (B) and (C) summarise the pooled data (*n* = 19–24 cells, 3 experiments) for percentage mobile fraction and diffusion co-efficient *D* respectively. **p < 0.01 indicates a significant difference between control and NPY groups (Kruskal–Wallis and Dunn's post test).

One surprising FCS observation, given significant Y1 receptor and Y2H155P receptor internalisation, was that measurements of surface receptor particle concentration in all four cell lines were not significantly altered by NPY treatment (Fig. 3B). This might be explained by an overall increase in the fraction of mobile receptor complexes at the plasma membrane following agonist stimulation, as immobile particles (relative to the read time) generate no fluorescence fluctuations and are undetectable by FCS [12,13]. We therefore performed FRAP experiments (at 37 °C) on the lower plasma membrane of 293TR Y1sfGFP or Y16AsfGFP cells (Fig. 4). As Fig. 4B illustrates, a high proportion (approximately 80%) of surface Y1sfGFP receptors were mobile, as observed for β2-adrenoceptors expressed in HEK293 cells [42,43], and this proportion did not vary with NPY treatment or with 6A mutation. Clearly this does not support an overall change in immobile receptor fraction that might influence FCS membrane particle concentrations. However it is also difficult to eliminate this possibility entirely, because of the very different spatial scales over which FRAP and FCS techniques operate. For FRAP, the minimal circular bleached area used (1.4 μm^2^) is still at least 14 times larger than for FCS measurements. This influence was also evident in FRAP-derived measurements of *D* based on the fluorescence recovery half time. There was correspondence with the FCS analysis in the overall ability of NPY to decrease Y1sfGFP, but not Y16AsfGFP receptor mobility (Fig. 4C). However FRAP estimates of *D* for Y1 receptors, whilst within the range obtained for other GPCRs [41,43,44], were an order of magnitude lower than those obtained by FCS from the upper membrane of the same cell lines. We attribute this difference to the FRAP diffusion co-efficients representing Y1 receptor diffusion over a much greater area than for FCS, in which cytoskeletal and other factors restricting longer range diffusion become more apparent [44,47]. For example, a significant proportion of GFP-tagged NK2 receptors expressed in HEK293 cells exhibit highly confined diffusion within plasma membrane domains of < 0.5 μm radius [44].

### 3.3 Generation of novel sfGFP BiFC partners for detection of receptor–arrestin association

Complementary FCS and FRAP approaches both demonstrated a clear correlation between agonist effects on Y1 and Y2 receptor membrane mobility, and an overall ability to undergo β-arrestin dependent internalisation. However diffusion co-efficients are still influenced by complex underlying molecular mechanisms. In FCS for example, a single measurement of *D* may reflect a heterogeneous ensemble of receptor complexes passing through the confocal volume, with differing molecular compositions. Moreover both particle size and kinetic parameters can determine overall mobility of individual receptor complexes. Mobility is relatively insensitive to mass, with *D* predicted to change by the cube root of molecular weight increases [12]. Although FCS is insensitive to immobile complexes over the time of the read, transient receptor interactions with slowly moving structures (for example clathrin coated pits), that are short relative to the measured dwell time, will also reduce observed *D* [12,13,21].

Improved interpretation of changes in *D* requires strategies that can help identify the actual molecular complexes being measured. Two colour FCS, in which fluctuations measured by two fluorescent protein partners are cross-correlated, provides one option, but is challenging to execute for membrane protein–protein signalling complexes [48]. An alternative approach is to use BiFC techniques to identify particular complexes [26,27]. The complemented fluorescence protein unambiguously identifies the protein–protein interaction, with the generated BiFC complex essentially behaving as a conditional fusion protein once formed. The fluorescence properties of the refolded split fluorescence protein are indistinguishable from the full length variant, and so enable its use in standard FCS experiments to study defined multi-protein complexes [18,26,27,49,50].

Previously we have shown that FCS in combination with BiFC, using well characterised YFP fragments, can investigate the distinct diffusion characteristics of adenosine receptor dimers of known identity [18]. However the use of YFP compared to GFP in FCS presents experimental limitations related both to an enhanced photophysical τ_D_ component, and to increased spot bleaching of fluorescent particles as they pass through the confocal volume [12]. Such spot bleaching reduces measured dwell times, and generates a faster observed *D* [28]. The significance of this effect was evident in our early experiments using unstimulated Y1 receptor-venus YFP in HEK293 cells (*D* 15.0  ± 1.1 × 10^− 9^ cm^2^/s, *n* = 8 — compare sfGFP and eGFP measurements in Table 2).

Whilst a GFP based BiFC system would be preferable for FCS experiments, split eGFP fragments show relatively poor complementation in living cells under physiological conditions [50]. In contrast the sfGFP variant substantially improves folding kinetics into the 11 strand β-barrel required for chromophore maturation [30], and BiFC assays have been developed which employ fragments split between β-strands 10 and 11 of sfGFP [51]. However these constructs exhibited spontaneous association and were not designed to be driven by association of tagged protein complexes. Thus we designed a new set of sfGFP BiFC partners (Gn, Gc), with the split point replicated from previous studies using overlapping BiFC YFP fragments [24,26,27,50] — Gn contained β-strands 1–8 of sfGFP (2–172), and Gc β-strands 8–11, sfGFP (155–238). We then examined the ability of these fragments to report the specific association between Y1 receptors (fused at the C terminus to Gc), and arrestin (tagged at the C terminus to Gn) — an assay we have previously described in depth for the YFP BiFC system [24].

We generated stable HEK293 cell lines expressing both Y1-Gc and either β-arrestin1-Gn (Y1 A1), β-arrestin2-Gn (Y1 A2) or β-arrestin2ΔLIEFD-Gn (Y1 A2ΔLIEFD). The targeted deletion of the L_373_IEFD_377_ in β-arrestin2 removes one binding motif for clathrin, and prevents this interaction in pull down assays [3]. C tail fusion of fluorescent protein fragments did not alter the ability of the Y1-Gc constructs to bind agonists with high affinity, or interact with G proteins, as assessed by GTPγS sensitivity of [^125^I]PYY binding to membrane preparations (Table 1), as previously reported [24]. Receptor expression levels ([^125^I]PYY B_max_ estimates) were also similar in Y1 A1, Y1 A2, and Y1 A2ΔLIEFD cell lines (Table 1).

**Fig. 5:**
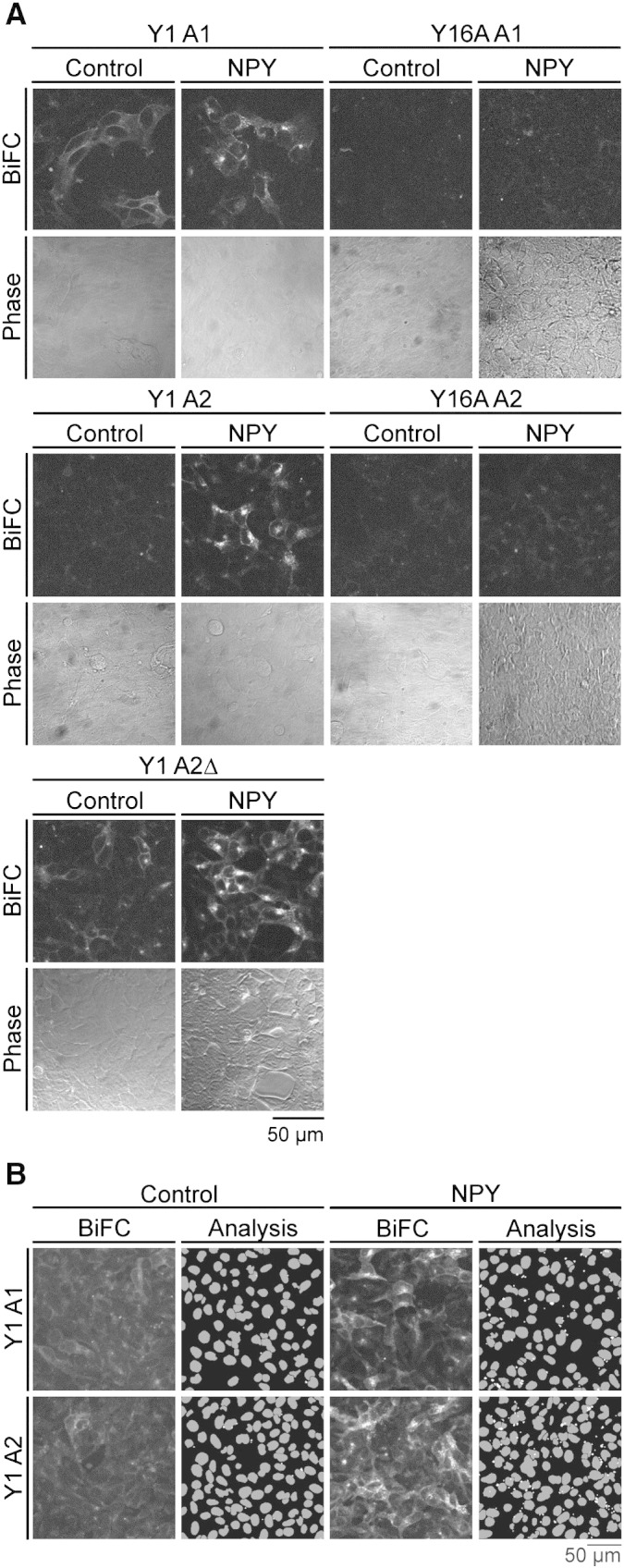
Identification of Y1 receptor β–arrestin association using sfGFP BiFC. Live HEK293 cells stably co-expressing Y1 receptor-Gc and either β-arrestin1-Gn (Y1 A1), β-arrestin2-Gn (Y1 A2) or β-arrestin2ΔLIEFD-Gn (Y1 A2Δ) were imaged by confocal microscopy at 37 °C (A). To the right, equivalent images are also shown for cells which instead expressed the mutant Y16A receptor-Gc in combination with β-arrestin-Gn (Y16A A1, Y16A A2). BiFC fluorescence was examined in cells under control conditions, or following 60 min 100 nM NPY treatment. Constant acquisition settings were used for the paired native and 6A mutant cell line images, taken during the same experiment (from n = 2–5). In B, images of the Y1 A1 and Y1 A2 cell lines were acquired using the IX Ultra confocal platereader, following vehicle or 100 nM NPY treatment and fixation. Representative examples are magnified to show 25% of the area of each original BiFC image. In each case the analysis panel shows the identification of nuclei (grey, original H33342 image not shown) and BiFC fluorescent compartments (> 3 μm diameter, white) by the granularity algorithm. This allowed measurement of average granule intensity/cell for each image, from which the quantified data in Fig. 6 was derived.

Fig. 5A shows resultant sfGFP BiFC fluorescence in living cells under control conditions, and following 60 min, 100 nM NPY treatment at 37 °C. For the Y1 A2 cell line, a minimal control background signal was observed. NPY stimulation generated new Y1 receptor-β-arrestin2 BiFC complexes, localised to intracellular perinuclear compartments with some labelling also present on the plasma membrane. Agonist dependent Y1 A2ΔLIEFD BiFC complexes were still internalised to intracellular compartments, despite deletion of the β-arrestin2 clathrin binding motif. This is consistent with additional arrestin binding motifs for both clathrin [6,7], and adaptor proteins such as AP-2 [3]. It suggests that this single mutation cannot completely eliminate functional endocytosis, which for the Y1 receptor proceeds in an arrestin and clathrin dependent manner [5,35,37]. Relatively more cell surface BiFC was observed, under both basal and agonist-treated conditions, in Y1 A2ΔLIEFD compared to Y1 A2 cells. Equally, whilst NPY also stimulated Y1 receptor / β-arrestin1 BiFC, some cell surface and intracellular pre-existing complexes were evident. We cannot rule out a contribution of non-specific “bystander” interactions to the formation of basal BiFC signals in each of these cell lines [26,27]. However no BiFC could be detected in equivalent β-arrestin1 and β-arrestin2 cell lines which coexpressed Y16A-Gc receptors (Fig. 5A, see also Table 1 for relative expression levels), under either control or agonist-stimulated conditions. This is also supported by quantitative data using the YFP BiFC system, in which basal Y1 receptor recruitment of β-arrestin1 and 2 was substantially reduced by the 6A mutation (Supplementary Fig. 1). Thus a significant element of the basal BiFC signal appears initiated by constitutive Y1 receptor recruitment of arrestin proteins (particularly β-arrestin1), supported by accumulating evidence for agonist independent GPCR–β-arrestin interaction [10,52].

**Fig. 6:**
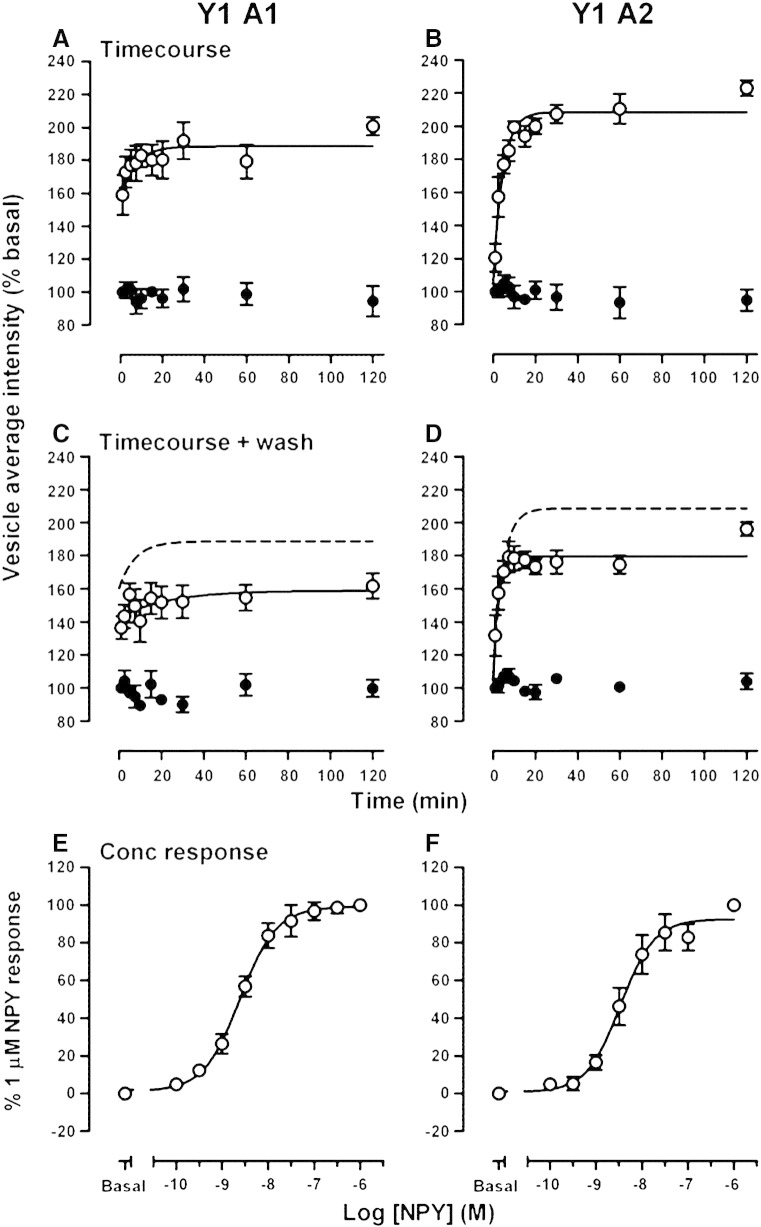
Timecourse, reversibility and concentration dependence of Y1 receptor β-arrestin association measured by sfGFP BiFC. Quantitative analysis was performed on automated plate reader images (see Materials and methods), to determine BiFC responses resulting from interaction of the Y1 receptor with β-arrestin1 (Y1 A1 cells; panels A, C, E) or β-arrestin2 (Y1 A2 cells; panels B, D, F). In A and B, 100 nM NPY timecourses (*n* = 4–5) were performed over 2 h at 37 °C (open circles), compared to vehicle controls (closed circles). Estimated half times from curve fitting (one phase association) are given in the text. In C and D, 100 nM NPY (solid symbols) timecourses were performed as before, together with vehicle controls (open symbols). Subsequently cells were washed with medium (2 × rinse, 1 × 60 min at 37 °C) to remove agonist, before fixation. NPY BiFC responses in the pooled data from treated and washed cells (*n* = 4–5) are compared with the original curve fits to the timecourse data without agonist removal (dotted line, from A or B). E and F show NPY concentration–response curves (*n* = 4) based on 60 min agonist treatment at 37 °C. pEC_50_ values are quoted in the text, and were very similar to those previously observed for YFP BiFC responses (Supplementary Fig. 1).

Y1 A1 and Y1 A2 BiFC complex development was also quantified by granularity analysis of automatically acquired confocal images from 96 well plates (Fig. 5B). 100 nM NPY stimulated BiFC responses, measured as the average vesicular intensity/cell, with t_1/2_ values of 3.0 ± 0.8 min (*n* = 4) and 5.2 ± 1.4 min (*n* = 5) for Y1 A1 and Y1 A2 cells respectively (Fig. 6A, B). These kinetics are similar to those for Y1 receptor β–arrestin2 association measured by YFP BiFC [24], suggesting equivalent rates of complementation for venus YFP and sfGFP BiFC fragments at 37 °C. As noted in our previous study, BiFC response kinetics do not reflect real time measurements of Y receptor–arrestin association, principally because of the delayed maturation of the GFP chromophore after refolding [24,26,49]. The faster kinetics in Y1 A1 cells are likely to be influenced by a contribution from rapid internalisation of pre-existing cell surface BiFC complexes at early timepoints (see Fig. 5A).

We also tested whether the sfGFP BiFC responses were reversible. Cells were treated with 100 nM NPY for differing times, as for the timecourse experiments. Thereafter the cells were washed extensively and left in media without agonist for 60 min at 37 °C before fixation. In the same HEK293 cell background, this protocol is sufficient for NPY removal and entirely reverses Y1 receptor-YFP internalisation [24]. However Fig. 6C and D illustrate that, whatever the initial time of agonist treatment, formation of endosomal BiFC complexes was largely irreversible in both Y1 A1 and Y1 A2 cells. Such irreversibility is an inherent characteristic of BiFC [24,26,27,49], demonstrating the formation of *de novo* receptor–β-arrestin1 and β-arrestin2 complexes following agonist stimulation.

NPY was equipotent in stimulating β-arrestin1 and β-arrestin2 recruitment (60 min incubation; Fig. 6E, F), with pEC_50_ values of 8.30  ± 0.10 (Y1 A1, *n* = 4) and 8.61 ± 0.10 (Y1 A2, *n* = 4). These estimates provide reliable measures of agonist efficacy in driving Y1 receptor–arrestin association, as they are unaffected by the choice of BiFC fragments used (compare Supplementary Fig. 1 and Ref. [24]), and are similar to potencies obtained using other assays of the receptor–β-arrestin pathway [39]. Our demonstration that Y1 receptors recruit both β-arrestin1 and β-arrestin2 to a similar degree (a “class B” GPCR phenotype [4]) is supported indirectly by the observation that activated Y1 receptors stimulate both β-arrestin1– and β-arrestin2–AP2 complexes measured by BRET [5].

### 3.4 Slow mobility of molecularly defined Y1 receptor–arrestin BiFC complexes

**Fig. 7:**
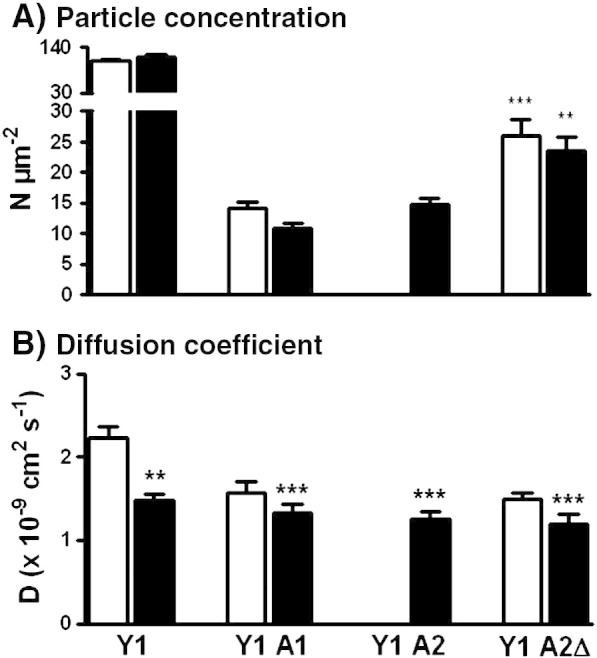
Diffusion of Y1 receptor — β-arrestin sfGFP BiFC complexes measured by FCS. FCS recordings were made from the upper membrane of Y1 A1 (control *n* = 21, 100 nM NPY treated *n* = 49 cells), Y1 A2 (NPY *n* = 64), and Y1 A2ΔLIEFD cells (Y1 A2Δ control *n* = 35, NPY *n* = 22), as described in the Materials and methods and illustrated in Fig. 2D. As previously, cells were pretreated at 37 °C for 60 min with vehicle (open bars) or NPY (solid bars), prior to FCS measurements at 22 °C. Pooled data is also compared with Y1sfGFP receptors (Fig. 3), acquired under identical acquisition conditions, for both particle concentrations (A, specific to component τ_D2_) and diffusion co-efficients derived from τ_D2_ (B). Plasma membrane fluorescence in Y1 A2 cells was negligible until these cells were stimulated with NPY, with no FCS possible under control conditions. Significant differences (**P < 0.01, ***P < 0.001; Kruskal–Wallis with Dunn's post test) refer to comparison with Y1 A2 NPY data (A) or Y1sfGFP control data (B).

Using the Y1 A1 and Y1 A2 BiFC cell lines we were able to correlate our observations on the slowed diffusion of NPY stimulated Y1 receptors, with the behaviour of the underlying molecular complex between the receptor and arrestin proteins. Y1 A1 and Y1 A2 cells were prestimulated with 100 nM NPY (60 min, 37 °C) to generate Y1 receptor–β-arrestin BiFC. The majority of these complexes were localised to intracellular compartments, but enough fluorescence was observed on the plasma membrane to enable FCS recordings to be made in the same way as for Y receptor–sfGFP fusion proteins (Fig. 2D). The same diffusional model was used, and sufficient, to analyse the resultant autocorrelation curves. Both the blinking component (data not shown), and the curve proportion and value of τ_D1_ were very similar for measurements based on full length or complemented sfGFP (Table 3) indicating equivalent fluorescent characteristics, at least for the purposes of FCS. There was an expected reduction in the observed particle concentrations for Y1 A1, Y1 A2 and Y1 A2ΔLIEFD cells (Fig. 7A), compared to the higher expression of Y1 receptor–sfGFP fusion proteins. Y1 receptor BiFC complexes containing either β-arrestin1 or β-arrestin2 both diffused with similar mobility (*D* 1.2–1.3 × 10^− 9^ cm^2^ s^− 1^; Fig. 7B and Table 3) to NPY-treated Y1 receptor-sfGFP proteins. Incorporation of the ΔLIEFD deletion into Y1 receptor–β-arrestin2 complex had no effect on *D*, consistent with the preserved internalisation of this BiFC complex (see Section 3.3). In FRAP analysis, the percentage of mobile Y1 A2 and Y1 A2ΔLIEFD particles (formed after 60 min NPY, 100 nM) showed no difference from Y1sfGFP measurements (80.0 ± 2.6% and 79.6  ± 2.6% respectively, *n =* 26–31; compare Fig. 4). These complexes also both displayed FRAP diffusion rates, equivalent to NPY-treated Y1sfGFP cells (*D* 0.63 ± 0.03 × 10^− 10^ cm^2^ s^− 1^ for Y1 A2 and 0.67 ± 0.08 × 10^− 10^ cm^2^ s^− 1^ for Y1 A2ΔLIEFD cells; *n* = 26–31).

FCS measurements were additionally possible for pre-formed Y1 A1 and Y1 A2ΔLIEFD BiFC complexes under basal conditions, which both displayed somewhat faster mobilities than after NPY treatment. However much reduced plasma membrane diffusion co-efficients were still observed compared to unstimulated Y1-sfGFP receptors (Fig. 7B, Table 3). This has parallels from other studies in which agonist-independent GPCR recruitment of β-arrestin2 was artificially induced, and shown to be sufficient, at least in part, for internalisation and downstream signalling [8,53]. Thus, even in the absence of agonist occupancy, Y1 receptor–arrestin complexes may adopt “active” conformations and scaffold interactions that dictate some of their diffusional characteristics.

### 3.5 PCH analysis reveals agonist-induced changes in the stoichiometry of Y1-sfGFP receptor complexes, and heterogeneity in Y1-β-arrestin particle composition

It has become apparent that GPCR signalling events may be moulded by receptor dimerisation, or the formation of higher order oligomers and clusters within a single signalling complex. Some of these changes are difficult to ascertain by standard FCS techniques, because the differences in molecular mass are too small to be reliably reflected in changes in diffusion coefficient (for example, the 2 fold transition from GPCR monomer to dimer leads to only a 1.25 fold change in *D*) [12,15]. The PCH statistical approach can instead analyse the same fluorescence fluctuation records with respect to amplitude, rather than time [15,16]. PCH divides the fluctuation record into bins of time *T* (chosen to be shorter than the dwell time of the diffusing species), and describes the resultant frequency distribution of photon counts (*k*) within these bins. Fluctuations in fluorescent light intensity are generated by the different excitation conditions experienced by fluorescent particles, as they diffuse through the confocal volume, and this creates deviations from an expected Poisson distribution for *k* [15]. This extent of these deviations depends on the particle number, *N*, and its molecular brightness ε, and thus these parameters can be derived, for single or multiple species, by modelling the PCH curve [15]. Molecular brightness measurements are very sensitive to the number of fluorescent molecules within a diffusing particle (with the change in GPCR monomer to dimer now leading to a 2 fold change in ε) and thus provide complementary information to FCS on the composition of diffusing fluorescent complexes.

**Fig. 8:**
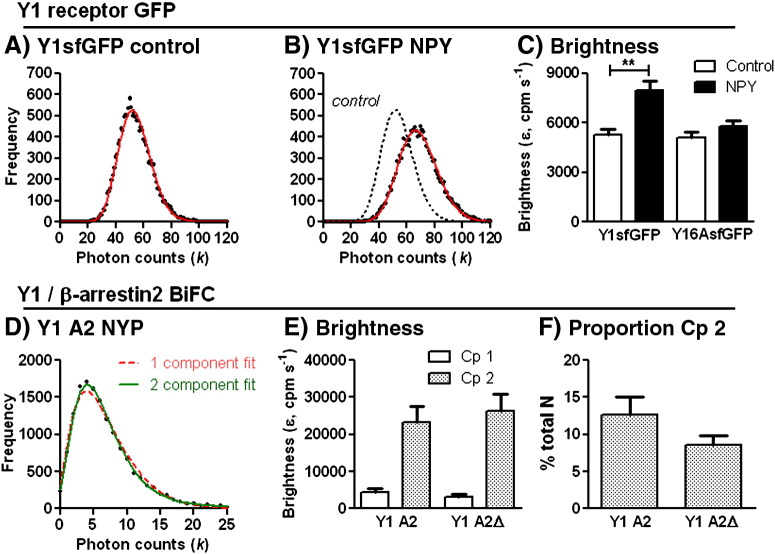
PCH analysis derives molecular brightness for Y1sfGFP and Y1-β-arrestin BiFC complexes. A and B illustrate representative one component PCH fits (red line) derived from single recordings of Y1sfGFP cells under control conditions or after NPY stimulation (from at least 3 experiments). Photon count frequency was calculated using a bin time set to 1 ms, from a 15 s read. The pooled data in C shows the effect of 100 nM NPY pretreatment on particle brightness (ε) derived for Y1sfGFP (*n* = 47–57) and Y16AsfGFP (*n* = 30–43) receptors. **P < 0.01 control *vs* NPY. Graph D demonstrates that a single component fit was not sufficient to model PCH data from diffusion of Y1-β-arrestin BiFC complexes in NPY treated Y1 A2 cells (red, analysis as A and B). Instead improved PCH fitting was performed with a two component model (green), with the proportion of each component assigned by the analysis. The two particle components (Cp 1, Cp 2) had markedly differing brightness (pooled data in E) for both Y1 A2 and Y1 A2ΔLIEFD (Y1 A2Δ) NPY treated cell data (3 experiments; *n* = 14–19). The proportion of very bright aggregated particles (Cp 2), as a percentage of the total is indicated in F, in which the difference between Y1 A2 and Y1 A2ΔLIEFD data groups was not significant (*p* = 0.051).

Fig. 8 illustrates PCH analysis applied to fluctuations recorded from 293TR Y1-sfGFP and Y1 A2 BiFC cells. Under both basal and 100 nM NPY treated conditions, photon counting histograms for Y1-sfGFP cell data (Fig. 8A, B) were generated by 1 ms bins, and described sufficiently by a single component PCH model. This analysis led to estimates of particle concentration of 350 ± 19 μm^− 2^ (control, *n* = 57) and 441 ± 35 μm^− 2^ (NPY, *n* = 47). Under control conditions, the brightness of these particles (Fig. 8C) was comparable to the brightness of GAPsfGFP (ε 5720 ± 621 counts per molecule (cpm) s^− 1^, *n* = 35) using the same one component analysis. As for measurements of Y1sfGFP, there was a consistent increase in derived particle concentration of GAPsfGAP from PCH analysis (633 ± 50 μm^− 2^, *n* = 35) compared to FCS (293 ± 32 μm^− 2^).

The similar brightness values obtained for Y1sfGFP and GAPsfGFP particles were initially surprising, given previous demonstration of Y1 receptor dimers by FRET [38] and similar analysis for μ-opioid receptor-GFP suggesting 1.5 hold higher brightness than free cytoplasmic GFP [20]. In retrospect, whilst a membrane-localised GFP marker provides a more appropriate reference point in principle for these comparisons, it presents complications when considering its particle composition. First the saturated lipid anchors may promote clustering of GAPsfGFP into membrane “raft” domains [31], with a resultant increase in the number of molecules per diffusing particle. Second high levels of expression, and restriction to a two dimensional membrane result in a concentration estimate for membrane GAPsfGFP of 0.05 mM (from FCS analysis). This concentration approaches the K_d_ for the formation of low affinity GFP dimers observed *in vitro* (0.1 mM) [31]. Thus, it is probably unsafe to assume the stoichiometry of GAPsfGFP is monomeric.

NPY treatment resulted in a significant 1.5 fold increase in Y1sfGFP particle brightness (Fig. 7C), indicating formation of particles containing more fluorescent receptors. This change was entirely prevented by 1 μM BIBO 3304 pre-incubation (brightness ε of 5412 ± 502 cpm s^− 1^ in cells treated with BIBO3304 alone *versus* 5740 ± 388 cpm s^− 1^ for BIBO3304 followed by 100 nM NPY, *n* = 24–28, 4 expts). One interpretation would be that agonist stimulation directly drives greater Y1 receptor oligomerisation as an inherent activation mechanism, and this has been suggested on the basis of similar analysis for opioid receptors [20]. However, whilst Y1 receptors form dimers detectable by FRET, the extent of dimerisation assessed by this method does not change with ligand occupancy [38]. Furthermore for the Y16AsfGFP receptor under basal conditions, PCH analysis indicated similar particle number (351  ± 29 μm^− 2^, *n* = 30) and brightness to Y1sfGFP receptor measurements. However the 6A mutation prevented any changes in particle brightness after NPY treatment (Fig. 8C; particle number 383 ± 30 μm^− 2^, *n* = 43). Given that it is unlikely that the 6A mutation alters Y1 receptor oligomerisation *per se*, these data suggest that increased overall brightness observed for NPY-stimulated Y1 receptor particles is instead an indirect consequence of β-arrestin dependent receptor clustering.

This conclusion was supported by PCH analysis for molecularly defined Y1-β-arrestin2 complexes (Fig. 8D–F). All histograms derived from Y1 A2 and Y1 A2ΔLIEFD recordings required a PCH fit with two components, rather than a single species (Fig. 8D). The predominant component displayed particle concentrations of 97.2 ± 20.3 μm^− 2^ (*n* = 18) for Y1 A2 and 97.6 ± 27.5 μm^− 2^ (*n* = 14) for Y1 A2ΔLIEFD after NPY, and showed similar brightness to Y1sfGFP particles under control conditions (*e.g*. Y1 A2 ε 4477 ± 1037 cpm s^− 1^, *n* = 18; *p* = 0.27 compared to Y1sfGFP control ε 5299 ± 291 cpm s^− 1^, *n* = 57). However PCH analysis also resolved a second component of 5–8 fold higher ε, representing aggregation of the BiFC complexes (Fig. 8E). The clustered component visible for Y1 arrestin BiFC complexes is thus linked to interaction with endocytotic machinery targeted by arrestin recruitment — most likely to be clathrin coated pits [2,3,5,35,37]. Uniquely, due to the size of the sampling confocal volume used for fluctuation analysis, it is possible to resolve these events to the immediate vicinity of the plasma membrane, on the cell surface or during initial endocytic vesicle formation. In addition the relatively large size of a single coated pit (~ 100 nm diameter), compared to the illuminated waist diameter under observation (~ 300 nm, Fig. 2A), suggests that multiple receptor–arrestin complexes may cluster within individual pits.

Aggregated complexes represented a significant proportion of “unit” Y1 A2 BiFC particles (~ 43% assuming a unit brightness of 4477 cpm s^− 1^). However the continued availability of single diffusing units, even in Y1 A2 cells, is consistent with the proposal that GPCR trapping in clathrin coated pits might be a low probability event [21], and also with the possibility of alternative GPCR–arrestin signalling complexes at the plasma membrane [1]. We should note that two distinct brightness components were only resolved in the Y1 A2, but not NPY treated Y1sfGFP histograms. It is likely that detection of multiple components in PCH analysis was enhanced with reduced particle concentration observed in BiFC cells [15]. In the Y1sfGFP system, a single ensemble component with higher overall brightness might still represent a similar mix of clustered and lower molecular weight complexes.

Comparison of our PCH and FCS results provides insight into the complementary nature, and some of the limitations of each technique. From the PCH data, very bright aggregates constituted a small proportion of Y1 A2 and Y1 A2ΔLIEFD particles. However, the mobility of these bright clustered complexes is likely to have the greatest influence on overall dwell times calculated by autocorrelation analysis of the time-dependent fluctuations, where the contribution made by individual particles is proportional to ε^2^ [12]. In our receptor systems, and for the membrane marker GAPsfGFP, we also observed a systematic, 2–3 fold, increase in estimates of *N* derived from PCH compared to FCS analyses. A number of potential explanations might account for this discrepancy. The first derives from the use of a 3D model for the PCH data, compared to the 2D diffusional model fitted to FCS autocorrelation curves. There are limits to the applicability of either model when considering the complex membrane environment of living cells, a 2D bilayer that nevertheless will include 3D topological features, such as coated pits. This may introduce sources of systematic error in absolute measurements obtained by the different techniques. It may be possible to explore this in future as new 2D PCH analysis methods are refined [54]. Second a contribution of the 150–250 μs τ_D1_ component to the overall PCH data cannot be entirely excluded, despite our choice of PCH bin time (1 ms) to prevent this. However this possibility is unlikely as repeating the PCH analysis with a much longer bin time (50 ms), did not significantly change overall *N* or ε obtained for the Y1sfGFP or Y1 A2 BiFC measurements (data not shown). Finally whilst both FCS and PCH techniques require particle diffusion to generate fluctuations – with respect to either amplitude or time – the threshold mobility for complexes to register in each analysis differs, and is lower for PCH. Thus it is likely that some slowly moving particles are counted by PCH, but do not influence FCS autocorrelation curves.

Unravelling the stoichiometry of receptor interaction with effectors, such as G proteins or β-arrestins, is a key challenge to advancing our understanding of GPCR pharmacology [55,56]. For β-arrestins a theoretical model of asymmetric binding to a GPCR dimer has been proposed, based on the two arrestin “sensor” domains each interacting with a different receptor protomer [57]. In this instance the formation of the high affinity agonist–GPCR–arrestin ternary complex might only stabilise ligand binding to one orthosteric site within a “unit” receptor dimer [55]. There are experimental studies which support this mode [58], but others have favoured a symmetric 1:1 (or 2:2) stoichiometry – for example for rhodopsin – visual arrestin interaction [59,60], or *in vitro* association between purified GPCRs and arrestins [61]. In our experiments the equivalent brightness of the non-clustered component for the Y1-β-arrestin complex and Y1sfGFP PCH data suggests the same number of GFP molecules per fluorescent complex, whether the receptor is labelled directly or via generation of receptor–β-arrestin BiFC. This is most simply explained by a symmetric mode of receptor–arrestin association — if binding were exclusively asymmetric, β-arrestin association would generate a single BiFC GFP molecule per two receptors, and an expected brightness of 0.5 ε compared to the equivalent Y1sfGFP complex. This conclusion is limited by an assumption that, other than clustering prior to endocytosis, the oligomeric state of Y1sfGFP receptors does not change significantly with agonist stimulation. Thus a potential distinction between symmetric GPCR-β-arrestin interaction, as suggested here, and the proposed asymmetric binding of GPCR dimers to the heterotrimeric G protein [62–64], needs further study. However confirmation could provide an interesting mechanism by which ligands, through their mode of binding to one or multiple orthosteric sites within a GPCR complex, are capable of “biased” activation of G protein or arrestin mediated signalling pathways [1,55,56].

## 4 Conclusions

FCS and PCH analyses are powerful approaches to investigate GPCR pharmacology at the single cell level, if relationships between the parameters measured and receptor function can be isolated. Here we have shown that these techniques can isolate changes in NPY receptor motility and oligomeric state following β-arrestin recruitment — measured at an early stage in the plasma membrane prior to endocytosis. For the first time our development of the novel GFP BiFC system enabled direct analysis of the molecular Y receptor–β-arrestin complex at the heart of this process and also yielded evidence for its symmetric stoichiometry. The improved photophysical characteristics of GFP BiFC will also provide future opportunities for using this technique in combination with FCS to dissect the function and composition of defined GPCR signalling complexes.

## Figures and Tables

**Table 1:** [^125^I]PYY binding data for Y receptor-sfGFP and BiFC HEK293 cell lines.

Cell line	PYY		NPY	GTPγS	
	pKi	B_max_ (pmol mg^− 1^)	pKi	pIC_50_	Inhibition (1 μM, %)
*sfGFP:*					
Y1	9.50 ± 0.23	1.5 ± 0.7	9.75 ± 0.16	9.01 ± 0.18	68.6 ± 6.7
Y16A	9.22 ± 0.09	2.3 ± 0.1	9.69 ± 0.04	8.80 ± 0.21	72.9 ± 7.0
Y2	10.76 ± 0.09	2.2 ± 0.3	10.39 ± 0.17	8.81 ± 0.14	69.2 ± 5.4
Y2H155P	10.57 ± 0.05	2.1 ± 0.2	9.91 ± 0.27	8.72 ± 0.15	46.9 ± 10.2

*BiFC:*					
Y1 A1	9.45 ± 0.19	0.7 ± 0.2	9.58 ± 0.07	8.62 ± 0.26	68.5 ± 13.9
Y1 A2	9.54 ± 0.06	0.9 ± 0.4	9.70 ± 0.13	8.54 ± 0.16	66.7 ± 7.0
Y1 A2Δ	9.44 ± 0.08	1.1 ± 0.3	9.76 ± 0.16	8.49 ± 0.02	54.9 ± 7.0
Y16A A1	9.30 ± 0.13	1.6 ± 0.2	9.76 ± 0.11	8.67 ± 0.12	69.4 ± 5.8
Y16A A2	9.48 ± 0.09	1.1 ± 0.3	9.73 ± 0.09	8.63 ± 0.02	78.2 ± 2.1

Data are presented as mean ± s.e.m. from 2 to 6 (typically 3) membrane competition binding experiments, using either 16 pM (Y1 receptor) or 10 pM (Y2 receptor) [^125^I]PYY as the radiolabel. Y1 A2Δ represents the Y1 receptor BiFC cell line incorporating the β-arrestin2 ΔLIEFD deletion mutant.

**Table 2:** Summary of FCS parameters for GFP tagged NPY receptors.

Receptor	τ_D1_	τ_D2_		*D*	n
	μs	%	ms	× 10^− 9^ cm^2^ s^− 1^	
*Y1eGFP*					
Control (5)	144 ± 5	46.3 ± 1.6	40.4 ± 2.2	2.05 ± 0.15	53
NPY (5)	190 ± 10	49.5 ± 2.1	59.3 ± 5.1	1.64 ± 0.15	46

*Y1sfGFP*					
Control (16)	250 ± 9	41.6 ± 1.1	40.0 ± 1.6	2.22 ± 0.15	148
NPY (16)	258 ± 9	39.6 ± 1.2	60.4 ± 3.4	1.48 ± 0.08	117

*Y16AsfGFP*					
Control (7)	230 ± 8	41.6 ± 1.4	33.9 ± 1.8	2.45 ± 0.12	87
NPY (7)	244 ± 8	42.6 ± 1.2	40.0 ± 2.0	2.09 ± 0.10	95

*Y2sfGFP*					
Control (4)	223 ± 11	52.2 ± 0.6	37.5 ± 2.2	2.15 ± 0.15	50
NPY (4)	265 ± 12	51.6 ± 0.6	46.4 ± 4.8	1.99 ± 0.15	47

*Y2H155PsfGFP*					
Control (4)	213 ± 11	53.4 ± 0.6	31.6 ± 2.1	2.55 ± 0.14	51
NPY (4)	260 ± 16	53.1 ± 1.3	53.0 ± 6.2	1.78 ± 0.16	35

Plasma membrane FCS measurements (2 × 15 s reads, with pre-bleach) were performed on stable transfected 293TR cells inducibly expressing Y receptors, under control conditions or treated with 100 nM NPY. Details are described in Materials and methods, and Fig. 2 provides illustrative examples. Autocorrelation curves were fitted with a two dimensional diffusional model with two dwell time components and a fluorophore blinking component (Zeiss Aim 4.2). The dwell times (mean ± s.e.m.) were attributed to GFP photophysics (τ_D1_) and receptor diffusion (τ_D2_). The proportion, % τ_D2_, refers to the percentage contribution of this component to the autocorrelation curve amplitude, relative to τ_D1_. *D* was calculated from τ_D2_ measurements from each individual record, before averaging the pooled data shown here. Y1 or Y2 receptor constructs were fused to constructs labelled with sfGFP or enhanced eGFP. *n* values quoted represent the number of cell records, whilst values in parenthesis to the left indicate the number of experiments performed.

**Table 3:** Summary of FCS parameters for Y1-arrestin complexes detected by BiFC.

BiFC complex	τ_D1_	τ_D2_		*D*	*N*
	μs	%	ms	(× 10^− 9^ cm^2^ s^− 1^)	
*Y1 A1*					
Control (3)	246 ± 20	52.1 ± 0.8	48.4 ± 3.7	1.57 ± 0.13	21
NPY (4)	279 ± 29	60.3 ± 1.4	72.3 ± 5.6	1.33 ± 0.10	49

*Y1 A2*					
NPY (9)	278 ± 17	63.3 ± 1.4	72.6 ± 6.2	1.26 ± 0.08	64

*Y1 A2Δ*					
Control (4)	259 ± 7	60.0 ± 1.6	48.8 ± 2.3	1.51 ± 0.08	35
NPY (4)	254 ± 11	57.8 ± 3.4	77.1 ± 7.0	1.20 ± 0.11	22

Fluorescence fluctuations were recorded (2 × 15 s reads) from dual stable HEK293 cells co-expressing Y1 receptor-Gc and β-arrestin-Gn BiFC partners. The development of BiFC complexes was stimulated by 60 min pre-treatment with 100 nM NPY (see Materials and methods, and Fig. 2). Values in parenthesis beside the treatment conditions indicate the number of experiments (no FCS measurements could be taken under control conditions for Y1 A2 cells), whilst *n* values denote the number of cell recordings. As before, autocorrelation curves were fitted with a two dimensional diffusional model with two dwell time components and a fluorophore blinking component (Zeiss Aim 4.2 software). Pooled data (mean ± s.e.m.) is given for dwell times τ_D1_ and τ_D2_, the percentage contribution of the τ_D2_ component to the autocorrelation curve amplitude, and derived diffusion co-efficient (*D*). Y1 A2Δ represents the Y1 A2ΔLIEFD cell line.

## Appendix A Supplementary data

Supplementary material.

Video 1 Internalisation of Y1sfGFP receptors in response to NPY (100 nM, 10 min).

Video 2 Y16AsfGFP receptors do not internalise after NPY (100 nM, 10 min).
