# Supplementary material for: Fluorescence correlation spectroscopy, combined with bimolecular fluorescence complementation, reveals the effects of β-arrestin complexes and endocytic targeting on the membrane mobility of neuropeptide Y receptors
Source: Biochim Biophys Acta. 2012 Jun;1823(6):1068–81. doi: 10.1016/j.bbamcr.2012.03.002 (PMC3793875; doi:10.1016/j.bbamcr.2012.03.002)
Supplement: Supplementary file 1 — Supplementary material. [file mmc1.pdf]

Fluorescence correlation spectroscopy, combined with bimolecular fluorescence complementation, reveals the effects of β-arrestin complexes and endocytic targeting on the membrane mobility of Neuropeptide Y receptors

Laura E. Kilpatrick, Stephen J. Briddon and Nicholas D. Holliday

Supplementary Figure 1

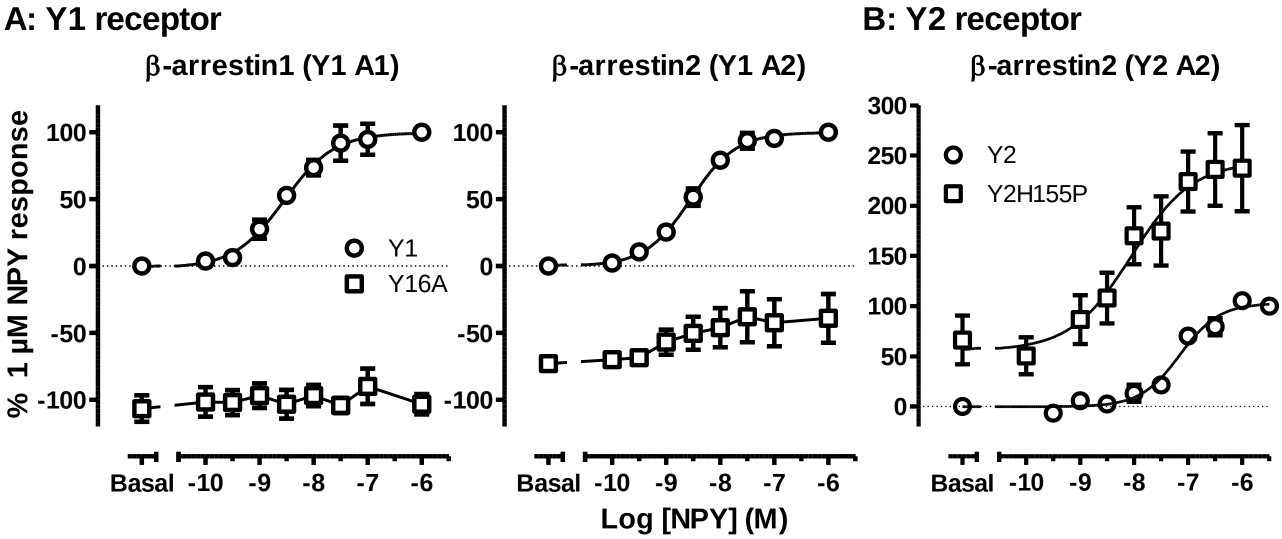

Supplementary Figure 1. Effect of the Y1 6A and Y2 H155P mutations on β-arrestin recruitment, assessed by YFP BiFC. Experiments were performed in HEK293 cells stably co-transfected with Y receptor-Yc and β-arrestin-Yn. [^{125}I]PYY B_{max} values were 340 - 350 fmol mg^{-1} for Y1 receptor and 1190 - 1440 fmol mg^{-1} for Y2 receptor expressing cells [1]. Cells were stimulated with NPY for 60 min, and YFP BiFC responses were quantified by automated platereader imaging and granularity analysis (see Materials and Methods). Pooled concentration response curves (*n* = 4 - 10) demonstrating the effects of the 6A mutation on NPY stimulated Y1 receptor β-arrestin2 BiFC (Y1 A2, panel A), and the H155P mutation on Y2 receptor β-arrestin2 BiFC (Y2 A2, panel B) have been reported previously [1]. They are repeated here for comparison with the Y1 receptor β-arrestin1 responses (Y1 A1, panel A), and with the receptor endocytosis data presented in Fig. 1. NPY pEC_{50} values were 8.54 ± 0.11 (Y1 A1, *n* = 4), 8.54 ± 0.07 (Y1 A2, *n* = 10), 7.15 ± 0.08 (Y2 A2, *n* = 5) and 8.05 ± 0.34 (Y2 A2, *n* = 5).

Supplementary Figure 2

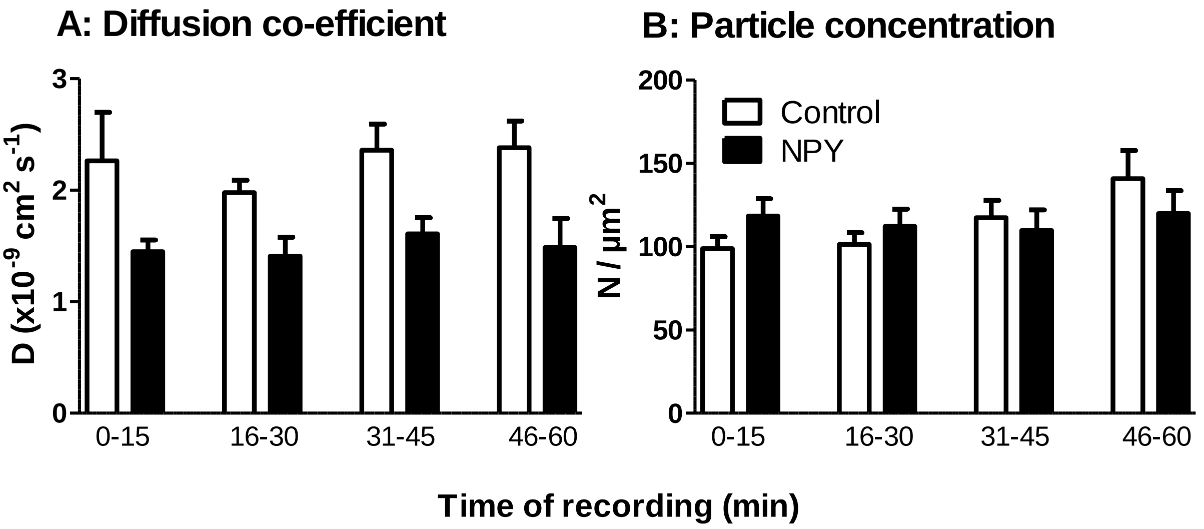

Supplementary Figure 2. Observed particle concentrations and diffusion co-efficients for Y1sfGFP receptors do not depend on the time of recording following vehicle or NPY vehicle pre-treatment. As outlined in the Materials and Methods, 293TR Y1sfGFP cells were incubated with vehicle or 100 nM NPY at 37°C for 15 min, after which they were equilibrated at room temperature for FCS measurements, made over the subsequent 60 min. The histograms here show mean ± s.e.m. values for diffusion co-efficients (A) and particle concentration (B) for cells binned according to the time of recording after the start of the room temperature incubation. The numbers of cell measurements per bin were 0-15 min (n = 40 - 64), 16 - 30 min (n = 35 - 39), 31 - 45 min (n = 29 - 32), 46 – 60 min (n = 11 - 14). Pooled data from the same experiments are presented in Fig. 3 and Table 2.

Supplementary Figure 3

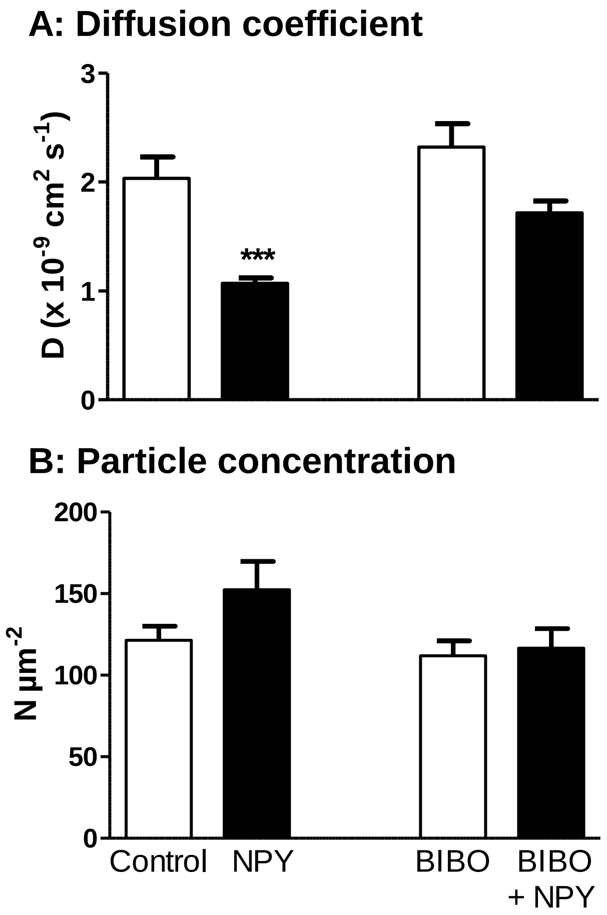

Supplementary Figure 3. The Y1 receptor antagonist inhibits changes in membrane Y1sfGFP receptor mobility measured by FCS. Histograms summarise the pooled data for diffusion co-efficients (*A*) and particle concentrations *(B)* from paired FCS experiments in 293TR cells induced to express Y1sfGFP (n = 20 - 29 cells, 4 expts). Following 15 min BIBO3304 (BIBO, 1 µM) pretreatment as appropriate, cells were incubated for 15 min with vehicle (control, open bars) or NPY (100 nM, solid bars) at 37°C. FCS recordings were made subsequently at room temperature. Significant differences between control and NPY treated groups are indicated by *** p < 0.001 (Kruskal-Wallis, followed by Dunn's post test).

Reference

[1] L.E. Kilpatrick, S.J. Briddon, S.J. Hill, N.D. Holliday, Quantitative analysis of neuropeptide Y receptor association with beta-arrestin2 measured by bimolecular fluorescence complementation, Br J Pharmacol, 160 (2010) 892-906.
